# Optimal Nutritional Support Strategy Based on the Association between Modified NUTRIC Score and 28-Day Mortality in Critically Ill Patients: A Prospective Study

**DOI:** 10.3390/nu15112465

**Published:** 2023-05-25

**Authors:** Sunny Park, So Hyang Park, Yeju Kim, Geon Ho Lee, Hyung-sook Kim, Sung Yoon Lim, Soo An Choi

**Affiliations:** 1 College of Pharmacy and Research Institute of Pharmaceutical Sciences, Korea University, Sejong 30019, Republic of Korea; psunny0708@korea.ac.kr; 2 College of Pharmacy, Korea University, Sejong 30019, Republic of Korea; 3 Department of Pharmacy, Seoul National University Bundang Hospital, Seongnam 13620, Republic of Korea; 4 Division of Pulmonary and Critical Care Medicine, Department of Internal Medicine, Seoul National University College of Medicine, Seoul National University Bundang Hospital, Seongnam 13620, Republic of Korea

**Keywords:** critically ill patients, 28-day mortality, modified NUTRIC score, nutrition screening tool, nutritional support strategy

## Abstract

Malnutrition in critically ill patients is closely linked with clinical outcomes. During acute inflammatory states, nutrition cannot reverse the loss of body cell mass completely. Studies on nutritional screening and strategy considering metabolic changes have not yet been conducted. We aimed to identify nutrition strategies using the modified Nutrition Risk in the Critically ill (mNUTIRC) score. Nutrition support data, laboratory nutrition indicators, and prognosis indices were prospectively collected on the 2nd and 7th day after admission. It was to identify the effect of the changes on the metabolic status and critical target of nutrition intervention. To discriminate the high-risk group of malnutrition, receiver operating characteristic curves were plotted. Risk factors associated with 28 day-mortality were evaluated using multivariable Cox proportional hazards regression. A total of 490 and 266 patients were analyzed on the 2nd and 7th day, respectively. Only the mNUTRIC score showed significant differences in nutritional risk stratification. The use of vasopressors, hypoprotein supply (<1.0 g/kg/day), high mNUTRIC score, and hypoalbuminemia (<2.5 mg/dL) in the recovery phase were strongly associated with a 28-day mortality. The implementation of the mNUTRIC score and protein supply in the post-acute phase is critical to improve 28-day mortality in critically ill patients.

## 1. Introduction

Malnutrition is highly prevalent in patients treated in the intensive care unit (ICU) and varies from 39–50% depending on screening tools and patient groups [1]. Poor nutritional status in critically ill patients is closely associated with negative clinical outcomes, such as a prolonged ICU stay, increased mortality, and infectious complications [2,3,4]. Therefore, adequate nutritional support is an essential component in the management of critical illness, and this should start with the identification of poor nutritional status of patients in the ICU [5].

Over the past decade, nutritional screening and assessment have become an integral part of nutrition care, with a variety of tools and guidelines available to healthcare professionals [6,7,8,9]. Nutritional risk screening 2002 (NRS-2002) has been shown to have good predictive validity in various hospitalized patients, but conflicting views have been shown in critically ill patients [10]. Since Heyland et al. introduced a severity index in the Nutrition Risk in the Critically Ill (NUTRIC) score [11], the modified-NUTRIC (mNUTRIC) score was developed during the studies on critically ill patients and has been validated across many observational studies from different countries [11,12,13,14]. However, the mNUTRIC score does not include traditional variables of nutrition, such as changes in food intake or body weight. In addition, studies regarding nutritional therapy using an mNUTRIC score are relatively lacking on Asians. 

The nutritional status of patients admitted to the ICUs deteriorates rapidly even in the case of well-nourished patients [4]. In fact, altered metabolism in critically ill patients exacerbates malnutrition, and the effects of inflammation on the nutritional status of patients is known [15]. During acute inflammatory states, nutrition cannot reverse the loss of body cell mass completely. These conditions predispose critically ill patients to a high risk of malnutrition [16]. For critically ill patients who are expected to stay in the ICU for more than 48 h, providing early nutrition is recommended as the standard of care [10,17]. Several studies have reported that nutritional adequacy, such as total calorie and protein intake during the first week in ICU, improved prognosis. This included reduced mortality and a shortened length of stay in the ICU [18,19,20,21]. Recently, the adequacy of nutritional supply has been evaluated according to the risk of malnutrition based on the mNUTRIC score [22,23]. When examining the pathophysiology of malnutrition across two main characteristics of critically ill patients, stress catabolism and inadequate nutritional intake, it is necessary to establish a nutrition screening strategy that considers metabolic changes in critically ill patients. However, such studies have not yet been conducted.

This study aimed to (1) assess the use of the mNUTRIC score compared to traditional screening tools in ICU patients, (2) evaluate the proper time to apply the mNUTIRC score to consider the metabolic characteristics of acute and recovery phases, and (3) identify critical nutrition strategies for improving 28-day mortality in the ICU.

## 2. Materials and Methods

### 2.1. Study Design and Patient Enrollment

This prospective observational study included all adult patients (aged ≥ 18 years) eligible for nutritional screening within two days of medical ICU admission at the Seoul National University Bundang Hospital from September 2020 to February 2022. The exclusion criteria were as follows: patients who were not eligible for nutritional screening within 48 h of ICU admission due to death, transfer, insufficient data, and discharge or by the judgment of the attending physician. All data collection and analysis procedures were approved by the Institutional Review Board of the Seoul National University Bundang Hospital (No. B-2009-634-301). Informed consent was obtained from all the participants or their respective guardians.

### 2.2. Nutrition Screening Tools and Data Collection

In all patients, basic patient information was explored at the time of ICU admission. The components necessary for nutrition screening and prognosis indices were collected from electronic medical records. Data of the following characteristics were recorded: age, sex, body weight, body mass index (BMI), source of ICU admission, diagnosis in the ICU, severity index (acute physiology and chronic health evaluation II (APACHE II), sequential organ failure assessment (SOFA)), use of renal replacement therapy (RRT), vasopressors, antibiotics, laboratory data (lymphocyte, white blood cell (WBC), albumin, C-reactive protein (CRP), and lactate), and 28-day mortality [24]. Variables to obtain scores for nutrition screening tools, such as reduced dietary intake and weight loss over a period of time and number of comorbidities, were observed in medical records or after interviewing patients or family members. Nutritional support data included total calories, proteins, and route of administration. Energy intake included calories received from enteral nutrition (EN), parenteral nutrition (PN), propofol, and intravenous glucose (>500 mL/day) infused hydration plus mixed fluid medication. The protein intake included EN and PN sources. To identify the impact of nutritional support, the achieved energy and protein levels were compared in two categories (hypocaloric and hypoprotein supply). Hypocaloric or underfeeding was defined as below 50% and 70% of the target energy calculated by the Harrison–Benedict equation on days 2 and 7, respectively [10,25]. In addition, patients consuming less than 1.0 or 1.3 g/kg/d were defined as the hypoprotein group [26,27]. The ideal body weight and actual body weight (if obese, adjusted body weight) were used for the nutritional supply of protein and calories provided to the patient, respectively [10]. Since this was an observational study, no attempt was made to change nutrition practices. All nutritional calculations were crosschecked by two authors.

For the comparison of nutrition screening tools, the NRS-2002, short Form of Mini Nutritional Assessment (MNA-SF), and mNUTRIC score, which have been studied in an ICU setting, were used. To compare the prognosis-related performance of nutritional risk screening tools under the same conditions, the MNA-SF was dichotomized into two groups (low and high risk for malnutrition) [28]. To identify the effect of changes in metabolic status of critically ill patients, all data were collected twice, first within 48 h of ICU admission (day 2 as the acute phase, observation range within 36–48 h) and 7 days after admission (day 7 as the recovery phase, observation range within 156–168 h after admission). 

### 2.3. Statistical Analysis

Statistical analyses were performed using the SAS software version 9.4. Demographic characteristics were described using Student’s *t*-test for continuous variables and chi-squared test for categorical variables. Continuous and categorical variables were summarized as mean ± standard deviation (median, interquartile range) and counts (percentile, %), respectively. Prognostic performance for predicting 28-day mortality among nutrition screening tools was compared using the area under the Receiver Operating Characteristic curve (ROC) by a logistic procedure. To evaluate sensitivity and specificity, the risk levels on days 2 and 7 were dichotomized according to nutritional status (high vs. low risk). The survival curves for 28-day mortality were derived using the Kaplan–Meier method, and the log-rank test was used for statistical comparison between high- and low-risk groups. The risk factors associated with the 28 day-mortality were evaluated using univariable and multivariable Cox proportional hazards regression. Covariates included basic demographics and different or significant factors on day 2 and 7, respectively. Multicollinearity among variables was tested, and multivariable regression was carried out based on the results of the univariable analyses. The final multivariable regression model was developed based on backward elimination. All statistical tests were two-sided, and the *p*-value of <0.05 was considered statistically significant.

## 3. Results

**Figure 1:**
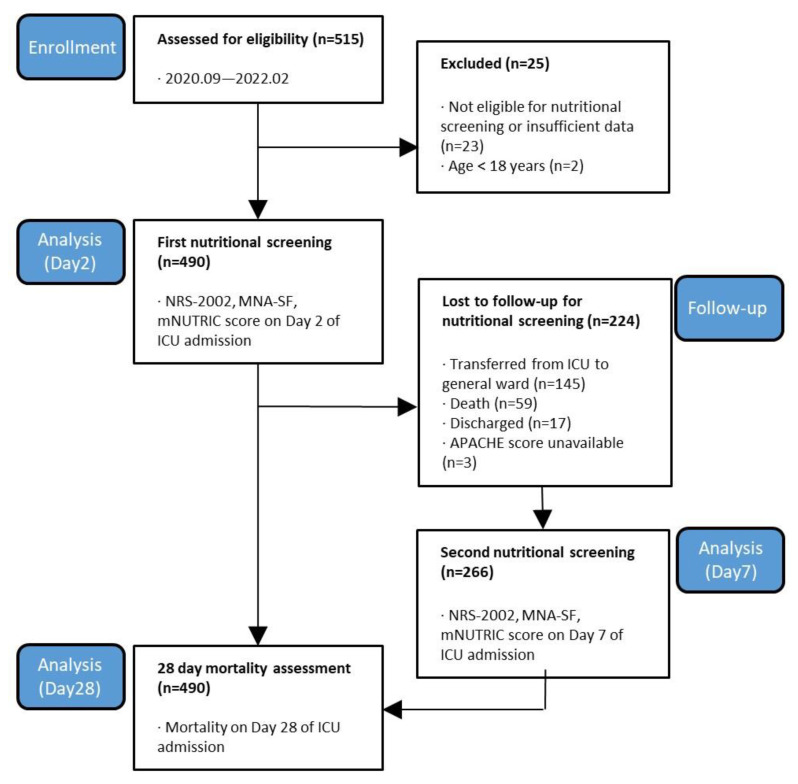
Flowchart of the study plan of patients selected for analysis.

Of the 515 patients admitted to the ICU during the study period, 490 patients were included on day 2, and we observed the prognosis, such as the 28-day mortality in all of them. Finally, 266 patients who were believed to be in the post-acute (recovery) phase were evaluated on day 7 for the second implementation phase of nutritional screening. A flow diagram of patient selection is shown in Figure 1.

The baseline characteristics of the enrolled patients according to the timing of nutritional screening are presented in Table 1. The study population was predominantly male (64.7%), with a mean age of 67.9 (±15.0) and BMI 23.6 (±5.6). Sex, age, BMI, ICU admission sources, days from hospital to ICU, number of comorbidities, and 28-day mortality did not differ significantly between the two groups. The most common diagnoses at admission were respiratory and circulatory diseases and neoplasms. Significant differences between days 2 and 7 were observed in the APACHE and SOFA scores, use of vasopressors, and routes of nutrition administration. Severity scores were lower on day 7 than on day 2 according to the mean APACHE score (from 28.6 on day 2 to 17.1 on day 7, *p* < 0.001) and mean SOFA score (from 7.5 on day 2 to 6.8 on day 7, *p* = 0.012). However, the high risk as per the mNUTRIC score on day 7 was still 51.5%. Nothing by mouth (NPO) patients decreased on day 7, and availability of nutritional support through various routes increased both total caloric and protein supply. Hypocaloric feeding patients on day 7 decreased from 61% to 37.2%, but protein supply below 1.0 g/kg was still 62.4%.

**Figure 2:**
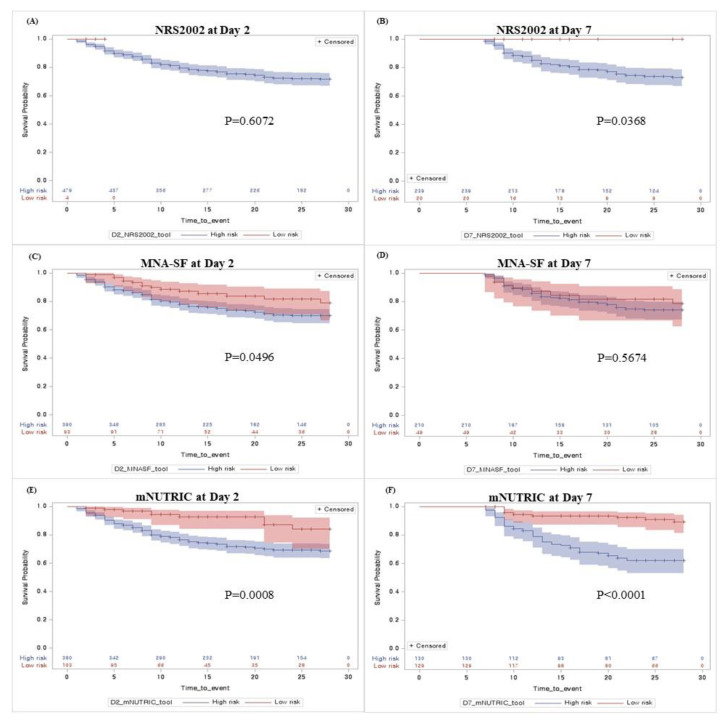
Kaplan–Meier survival curves obtained by nutrition screening tools in ICU patients.

**Figure 3:**
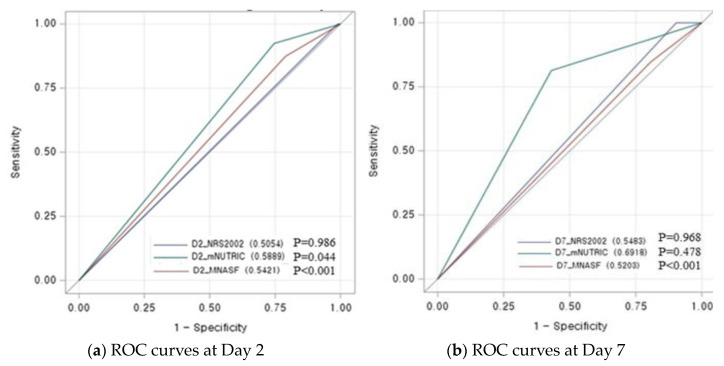
Receiver Operating Characteristic curve (ROC) curves to predict 28-day mortality in ICU patients using nutrition screening tools on Day 2 and 7.

Here, Figure 2 shows the Kaplan–Meier survival function for the risk group stratified by NRS-2002, MNA-SF, and mNUTRIC scores on days 2 and 7, respectively. The NRS-2002 classified most patients as having a high risk of malnutrition, even on ICU day 7. Only the mNUTRIC score showed significant differences in nutritional risk stratification on days 2 and 7. We also analyzed the differences between survivors and non-survivors at 28 days from ICU admission (Supplementary Materials). All patients, with or without survival, had a high mNUTRIC score at day 2, and the survivor group has reduced a markedly mNUTRIC score at day 7 (*p* < 0.001). In addition, comparison of the 28-day mortality prediction with nutrition screening tools using ROC analysis showed a good predictive value for the mNUTRIC score and was performed on day 7 (0.692, CI: 0.631–0.752, *p* < 0.001, Figure 3). 

**Figure 4:**
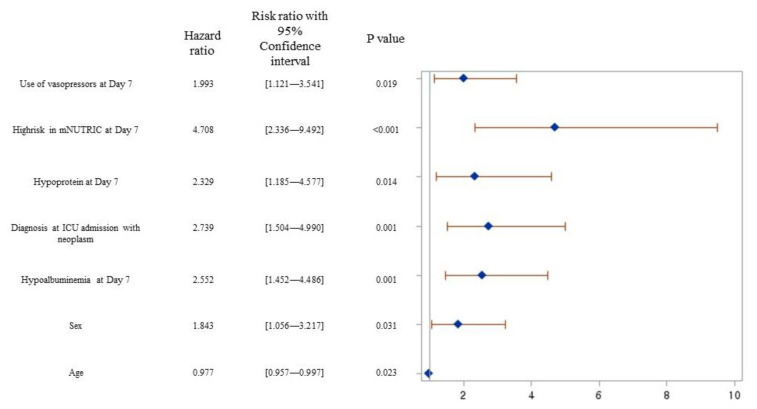
Adjusted hazard ratios of significant variables for 28-day mortality.

Using univariate analysis as the first step to affirm the risk factors, total calorie and protein amount, hypoalbuminemia (<2.5 mg/dL), neoplasm, renal dialysis, use of vasopressors, hypocaloric supply, hypoprotein (<1.0 g/kg, only on day 7), and mNUTRIC score (low and high risk) were identified as significant covariates that influenced 28-day mortality. Finally, the multivariate Cox proportional hazards regression showed that patients with a neoplasm (adjusted hazard ratio, aHR = 2.739, CI: 1.504–4.990, *p* = 0.001) and use of vasopressors on day 7 (aHR = 1.993, CI: 1.121–3.541, *p* = 0.019) were associated with a significantly higher 28-day mortality. Hypoalbuminemia (aHR = 2.552, CI: 1.452–4.486, *p* = 0.001) and hypoprotein supply (aHR = 2.329, CI: 1.185–4.577, *p* = 0.014) on day 7 also negatively influenced 28-day mortality as nutritional factors. In particular, patients assessed as having high risk according to mNUTRIC score on day 7 were predicted to have the poorest survival result (aHR = 4.708, CI: 2.336–9.492, *p* < 0.0001) (Figure 4).

## 4. Discussion

We aimed to identify critical nutrition strategies using the mNUTRIC score and predict a major prognosis, such as 28-day mortality in ICU patients. In addition, we sought to explore when it would be more appropriate to implement nutritional screening tools used in ICUs to reflect the patient’s metabolic state. In this study, the mNUTRIC score applying at ICU day 7 was shown to be better in predicting 28-day mortality compared with others. In addition, a high risk by mNUTRIC score, use of vasopressor, hypoprotein supply below 1.0 g/kg/day, and hypoalbuminemia (<2.5 mg/dl) in ICU patients going into a recovery phase were strongly associated with 28-day mortality.

### 4.1. Prognostic Performance of mNUTRIC Score for 28-Day Mortality

Previous studies have confirmed that a high mNUTRIC score is associated with poor clinical outcomes in ICU patients [13,22,29,30]. Our study results are similar to those of other studies. However, unlike studies involving NRS-2002, the mNUTRIC score was the only screening tool that showed validity in discriminating patients at high risk of 28-day mortality in our study [23,31,32]. In particular, NRS-2002 showed an unfair prediction for 28-day mortality (AUC at days 2 and 7, 0.505 and 0.548, respectively), which was significantly lower than that reported by Majari et al. (AUC 0.695) and Ma et al. (AUC 0.726) [31,32]. Although the NRS-2002 is recommended for use in various populations, one of the limitations of the use of this tool in ICU patients is related to the low cutoff value in terms of disease severity (APACHE II ≥ 10), which can lead to overestimation of high nutrition risk in the ICU [7,23]. In fact, because our study patients had much higher APACHE scores (median 29/IQR 22–35 at day 2) than previous studies (Majari et al., median 20/IQR 17–24 and Ma et al., median 14/IQR 10.5–18) [31,32], most of the patients were classified as high-risk groups of NRS-2002 (99.2%), and the survival analysis for 28-day mortality did not show significant results. Compared to NRS-2002, MNA-SF was not overly identified for nutritional risk (classified as high risk on day 2, 99.2% vs. 81%) but showed insignificant predictive performance for 28-day mortality (Figure 2). Since it includes parameters such as a history of recent weight loss or reduced food intake, this is inappropriate in critically ill patients on life support who are non-communicative and unable to provide such details. Therefore, the absence of classic nutritional variables, which is considered a major limitation of the mNUTRIC score, is more appropriate for predicting the prognosis and identifying the beneficial group of nutrition intervention through nutritional risk assessment in the ICU. Additionally, with increasing severity in ICU patients, the mNUTRIC score may help to better identify poor prognosis according to the risk of malnutrition than other screening tools.

### 4.2. Adequate Timing to Implement the mNUTRIC Score in Critically Ill Patients

The broad definition of nutritional screening focuses on the identification of patients who might be malnourished or are “at nutrition risk”. This simplifies the screening time at the time of hospitalization [33,34]. Most nutritional screening tools have been developed in outpatient or inpatient settings and do not include variables depending on the time of application [6,7]. In contrast, the mNUTRIC score integrates the severity of illness scores into its risk assessment calculations. Critically ill patients become metabolically/hemodynamically unstable during the acute phase, which is immediately after ICU hospitalization, within 5–7 days [10]. Therefore, we hypothesized that the timing of the mNUTRIC score for predicting prognosis would be more appropriate after the acute phase. Our results demonstrated that mNUTRIC on day 7 not only showed good predictive performance, but also exhibited a significant probability of 28-day mortality at high risk (aHR = 4.708, *p* < 0.0001). 

Even though the mNUTRIC score in the recovery phase was better than the acute phase, it was estimated less predictable than that of other studies (Heyland et al., AUC 0.783; Manon et al., AUC 0.768; and Majari et al., AUC 0.806) [11,13,31]. This difference can be explained as follows: first, the distinctive characteristic of the study subjects was that they had a higher average age (67.9 years) and severity scores than other studies. The period of this study corresponds to the period of the COVID-19 pandemic, and during this period, it was judged that the severity of the patient was higher than before due to limited ICU beds. Actually, the SOFA scores of our patients were much higher than those of studies conducted at tertiary hospitals of a similar size in Korea before COVID-19 [35]. Second, it may be due to differences in the time of data collection related to the nutritional screening tool. In other studies, data for nutritional screening were obtained within 24 h of admission to the ICU, whereas our study allowed 36–48 h. During the hyperacute early phase, the patient status is characterized by more severe metabolic instability and an increase in catabolism [10]. Thus, during our observation period, patients needed a more intensive treatment strategy. There existed a difference in the initial predicting ability due to the patient’s unstable condition.

### 4.3. Nutrition Support Strategy for Improving 28-Day Mortality

Inflammation during the acute ICU phase is usually associated with elevated CRP levels and hypoalbuminemia. A rapid loss of protein in ICU patients is most likely related to the proinflammatory state and severe catabolism due to an increase in stress-related cytokines and hormones. In one study, patients lost approximately 10~15% of their initial total protein content within 10 days of an ICU stay despite previous good nutritional status and adequate protein and energy intake [36]. Our study population, as shown by demographics, had improved CRP and APACHE II levels but displayed a decrease in albumin levels during the acute and recovery phases. When examining the nutritional support on ICU day 7, 62.8% of patients were supplied with ≥70% of calculated calories, but hypoprotein was still 62.4%. In addition, hypoalbuminemia and hypoprotein supply were significant factors as a negative influence on the 28-day mortality. This shows that supplementation with lost protein in the acute phase is a very important nutritional support strategy for improving the prognosis of patients who enter the recovery period. Recent reports indicate that higher nutritional adequacy evaluated in terms of calorie intake may reduce 28-day mortality in patients with a high mNUTRIC score [22,23]. The European Society of Parenteral and Enteral Nutrition guideline recommends that hypocaloric nutrition (below 70% estimated needs used predictive equation) should be preferred for the first week of an ICU stay [10]. We did not observe an association between hypocaloric feeding and 28-day mortality. As we applied strict calorie calculation, including those from dextrose fluid and propofol, the impact of a low calorie intake could have been further identified if the only considered calories were supplied through EN + PN as in other studies. It also seems that the severity of patients enrolled in our study may have offset the beneficial effects of caloric intake. Lastly, we used the Harris–Benedict equation as the predictive equation to calculate the hypocaloric nutrition. It could affect the different results regarding the calorie intake. Therefore, further studies are needed considering the difference between the resting energy expenditure or weight-based energy expenditure. 

### 4.4. New Insights and Limitations

This is the first prospective study of the mNUTRIC score considering the characteristics of critically ill patients who go through the acute and recovery phases in the ICU setting. Our results suggest that a nutritional intervention in those identified as a greater risk by the mNUTRIC score at the recovery phase have a benefit to the 28-day mortality. One of the limitations of our study is that it was conducted in a single center in Korea. In addition, the recovery period (ICU day 7) applied in our study was based on commonly suggested metabolic characteristics without objective measurements, such as inflammation indicators. Thus, it is thought that there will be actual differences in individual patients. Lastly, as this was a prospective observational study restricting any nutritional interventions, causality cannot be assumed. Therefore, further research is needed to compare the effects of aggressive nutritional support in patients who are identified to be at a high risk by the mNUTRIC score.

## 5. Conclusions

The implementation of the mNUTRIC score in the post-acute phase is the optimal time for considering metabolic characteristics. Hypoprotein intake (<1.0 g/kg/d) in post-acute phase patients with a high mNUTRIC score is associated with an increased risk of 28-day mortality.

## Figures and Tables

**Table 1:** Patient characteristics categorized according to nutritional screening day.

	Nutritional Screening Group on Day 2 (N = 490)	Nutritional Screening Group on Day 7 (N = 266)	*p* Value
Age (years)	67.9 ± 15.0	68.8 ± 14.5	0.408
Sex (N, %)			0.685
Male	317 (64.7%)	176 (66.2%)	
Female	173 (35.3%)	90 (33.8%)	
Body mass index (kg/m^2^)	23.5 ± 5.6	23.8 ± 6.6	0.509
Weight at ICU admission (kg)	62.1 ± 15.2	62.6 ± 17.4	0.673
Days from hospital to ICU (days)	5.4 ± 11.4 (1, 0–6)	6.9 ± 13.2 (1, 0–8)	0.110
Source of admission to ICU (N, %)			0.346
Ward	189 (38.6%)	104 (39.1%)	
Emergency room	254 (51.8%)	128 (48.1%)	
ICU	47 (9.6%)	34 (12.08)	
Comorbidities ≥ 2 (N, %)	411 (83.88%)	233 (87.59%)	0.170
APACHE II score	28.6 ± 8.9 (29, 22–35)	17.1 ± 7.8 (16, 12–22)	<0.001
SOFA score	7.5 ± 3.7 (8, 5–10)	6.8 ± 3.5 (6.5, 4–9)	0.012
Vasopressors (N, %)	335 (68.5%)	135 (51.1%)	<0.001
Renal dialysis (N, %)	120 (24.5%)	56 (21.1%)	0.286
Antibiotics (N, %)	408 (83.4%)	214 (80.5%)	0.304
Route of administration ^§^			<0.001
NPO	35 (7.2%)	3 (1.1%)	
EN	91 (18.7%)	68 (25.6%)	
PN	214 (43.9%)	54 (20.3%)	
EN + PN	148 (30.3%)	141 (53.0%)	
Calorie ENPN (kcal/day)	587.5 ± 505.4	1074.5 ± 589.8	<0.001
Hypocaloric ^§§^	299 (61.0%)	99 (37.2%)	<0.001
Protein Supply (g/kg/day)	0.4 ± 0.5	0.8 ± 0.5	<0.001
Hypoprotein (<1.0 g/kg/day)	441 (90.0%)	166 (62.4%)	<0.001
Hypoprotein (<1.3 g/kg/day)	463 (94.5%)	220 (82.7%)	<0.001
Diagnosis at ICU admission			0.734
Respiratory system	123 (25.1%)	74 (27.8%)	
Circulatory system	104 (21.2%)	56 (21.1%)	
Neoplasm	76 (15.5%)	37 (13.9%)	
Digestive system	38 (7.8%)	15 (5.6%)	
Infectious (Including COVID-19)	33 (6.8%)	23 (8.8%)	
Others	116 (23.7%)	61 (22.9%)	
Albumin (mg/dL)	2.9 ± 0.9 (2.8, 2.4–3.2)	2.7 ± 0.5 (2.7, 2.5–3.0)	0.013
CRP (mg/L)	11.6 ± 9.1 (9.0, 4.0–17.6)	8.5 ± 7.2 (6.3, 3.3–11.2)	<0.001
Lactate (mg/dL)	3.4 ± 4.2 (2.0, 1.3–3.3)	2.1 ±2.6 (1.4, 1.1–2.2)	<0.001
WBC (/mm^3^)	13.6 ± 12.6 (11.1, 7.8–15.7)	12.4 ± 8.0 (10.8, 7.5–15.2)	0.163
mNUTRIC			<0.001
Low risk	103 (21.0%)	129 (48.5%)	
High risk	387 (79.0%)	137 (51.5%)	
NRS2002			<0.001
Low risk	4 (0.8%)	20 (7.5%)	
High risk	486 (99.2%)	246 (92.5%)	
MNA-SF			0.851
Low risk	93 (19.0%)	49 (18.4%)	
High risk	397 (81.0%)	217 (81.6%)	
28-day mortality			0.515
Death (N, %)	119 (24.3%)	59 (22.2%)	

The variables are given in number (%) or mean ± standard deviation (median, Q25–Q75). Data were partially missing for albumin (1), CRP (25), lactate (102), WBC (2), vasopressors (1), and antibiotics (1). ICU; intensive care unit, NPO; Nothing per oral EN; Enteral Nutrition, PN; Parenteral Nutrition, CRP; C-reactive protein, WBC; white blood cell. ^§^ Two patients were discharged before nutritional support on day 2 after admission. Differences were observed between the EN-PN, EN-NPO, ENPN-PN, and ENPN-NPO. ^§§^ Hypocaloric intake was considered less than 50% of BMR on day 2 and 70% on day 7, respectively.

## Data Availability

Data is not available due to privacy and ethical restrictions.

## Supplementary Materials

The following supporting information can be downloaded at: https://www.mdpi.com/article/10.3390/nu15112465/s1, Table S1: Demographics and nutrition screening between survivors and non-survivors.
