# Supplementary material for: Optimal Nutritional Support Strategy Based on the Association between Modified NUTRIC Score and 28-Day Mortality in Critically Ill Patients: A Prospective Study
Source: Nutrients. 2023 May 25;15(11):2465. doi: 10.3390/nu15112465 (PMC10255887; doi:10.3390/nu15112465)
Supplement: Supplementary file 1 [file nutrients-15-02465-s001.zip › nutrients-2369820-supplementary.pdf]

Table S1. Demographics and nutrition screening between survivors and non-survivors

		Survivor at 28 day after ICU admission (N=371)	Non-survivor at 28 day after ICU admission (N=119)	P value
Age (years)		67.3± 15.3	69.6 ± 13.9	0.152
Sex (N, %)				0.340
	Male	244 (65.8%)	73 (61.3%)	
	Female	127 (34.2%)	46 (38.7%)	
Body mass index (BMI, kg/m^2)		23.5 ± 5.5	23.4 ± 5.8	0.798
Weight at ICU admission (kg)		62.6 ± 16.0	60.6 ± 12.1	0.222
Days from hospital to ICU (days)		4.7 ± 10.7	7.6 ± 13.4	0.039
Days of ICU admission (days)		12.3 ± 13.0 (8, 5-13)	8.5 ± 6.8 (7, 4-12)	0.002
Comorbidity ≥ 2 (N, %)		304 (81.9%)	107 (89.9%)	0.040
Source of admission to ICU (N, %)				0.134
	Ward	134 (36.1%)	55 (46.2%)	
	Emergency room	199 (53.6%)	55 (46.2%)	
	ICU	38 (10.2%)	9 (7.6%)	
APACHE II	Day 2	27.2 ± 8.5 (27, 21-34)	32.8 ± 8.8 (33, 28-39)	<0.001
	Day 7	16.2 ± 7.7 (15, 11-21)	20.3 ± 7.3 (20, 14-25)	<0.001
SOFA score	Day 2	6.8 ± 3.4 (7, 4-9)	9.5 ± 3.6 (10, 7-12)	<0.001
	Day 7	4.3 ± 3.4 (4, 2-7)	9.4 ± 3.7 (9, 7-12)	<0.001
Vasopressors	Day 2 (N, %)	236 (63.8%)	99 (83.2%)	<0.001
	Day 7 (N, %)	103 (29.5%)	41 (65.1%)	<0.001
Renal dialysis	Day 2 (N, %)	74 (20.0%)	46 (38.3%)	<0.001
	Day 7 (N, %)	43 (12.3%)	19 (30.2%)	<0.001
Diagnosis at ICU admission §				<0.001
	Respiratory system	97 (26.2%)	26 (21.9%)	
	Circulatory system	85 (22.9%)	19 (16.0%)	
	Neoplasms	41 (11.1%)	35 (29.4%)	
	Digestive system	29 (7.8%)	9 (7.6%)	
	Infectious (Including covid-19)	24 (6.5%)	9 (7.6%)	
	Others	95 (25.6%)	21 (17.7%)	
NRS2002 at Day 2				0.255
	Low risk	4 (1.1%)	0 (0%)	
	High risk	367 (98.9%)	119 (100%)	
NRS2002 at Day 7				0.013
	Low risk	20 (9.7%)	0 (0%)	
	High risk	187 (90.3%)	59 (100%)	
MNASF at Day 2				0.042

	Low risk	78 (21.0%)	15 (12.6%)	
	High risk	293 (79.0%)	104 (87.4%)	
MNASF at Day 7				0477
	Low risk	40 (19.3%)	9 (15.3%)	
	High risk	167 (80.7%)	50 (84.8%)	
mNUTRIC at Day 2				<0.001
	Low risk	94 (25.3%)	9 (7.6%)	
	High risk	277 (74.7%)	110 (92.4%)	
mNUTRIC at Day 7 [§§]				<0.001
	Low risk	118 (57.0%)	11 (18.6%)	
	High risk	89 (43.0%)	48 (81.4%)	

The variables are given in number (%) or mean ± standard deviation (median, Q25-Q75).
[§] The differences were observed between neoplasm-respiratory, neoplasm-circulatory, and neoplasm-digestive.
[§§] Day 7 was analyzed in 266 patients.
